# Post-load glucose subgroups and associated metabolic traits in individuals with type 2 diabetes: An IMI-DIRECT study

**DOI:** 10.1371/journal.pone.0242360

**Published:** 2020-11-30

**Authors:** Morgan Obura, Joline W. J. Beulens, Roderick Slieker, Anitra D. M. Koopman, Trynke Hoekstra, Giel Nijpels, Petra Elders, Robert W. Koivula, Azra Kurbasic, Markku Laakso, Tue H. Hansen, Martin Ridderstråle, Torben Hansen, Imre Pavo, Ian Forgie, Bernd Jablonka, Hartmut Ruetten, Andrea Mari, Mark I. McCarthy, Mark Walker, Alison Heggie, Timothy J. McDonald, Mandy H. Perry, Federico De Masi, Søren Brunak, Anubha Mahajan, Giuseppe N. Giordano, Tarja Kokkola, Emmanouil Dermitzakis, Ana Viñuela, Oluf Pedersen, Jochen M. Schwenk, Jurek Adamski, Harriet J. A. Teare, Ewan R. Pearson, Paul W. Franks, Leen M. ‘t Hart, Femke Rutters, for the IMI-DIRECT Consortium

**Affiliations:** 1 Epidemiology and Data Science, Amsterdam Public Health Research Institute, Amsterdam University Medical Centre, Location VU University Medical Center, Amsterdam, The Netherlands; 2 Julius Center for Health Sciences and Primary Care, University Medical Center Utrecht, Utrecht, The Netherlands; 3 Department of Cell and Chemical Biology, Leiden University Medical Center, Leiden, The Netherlands; 4 Department of Health Sciences, Faculty of Science, Vrije Universiteit Amsterdam, Amsterdam, The Netherlands; 5 Department of General Practice and Elderly Care Medicine, Amsterdam Public Health Research Institute, Amsterdam University Medical Centre, Location VU University Medical Center, Amsterdam, The Netherlands; 6 Department of Clinical Sciences, Genetic and Molecular Epidemiology Unit, Lund University, Malmö, Sweden; 7 Oxford Centre for Diabetes, Endocrinology and Metabolism (OCDEM), University of Oxford, Oxford, United Kingdom; 8 Department of Medicine, University of Eastern Finland and Kuopio University Hospital, Kuopio, Finland; 9 The Novo Nordisk Foundation Center for Basic Metabolic Research, Faculty of Health and Medical Sciences, University of Copenhagen, Copenhagen, Denmark; 10 Department of Cardiology and Endocrinology, Slagelse Hospital, Slagelse, Denmark; 11 Faculty of Health Sciences, University of Southern Denmark, Odense, Denmark; 12 Eli Lilly Regional Operations GmbH, Vienna, Austria; 13 Division of Cardiovascular & Diabetes Medicine, Medical Research Institute, University of Dundee, Dundee, United Kingdom; 14 Sanofi-Aventis Deutschland GmbH, R&D, Frankfurt am Main, Germany; 15 Institute of Biomedical Engineering, National Research Council, Padova, Italy; 16 Wellcome Trust Centre for Human Genetics, University of Oxford, Oxford, United Kingdom; 17 Institute of Cellular Medicine (Diabetes), Newcastle University, Newcastle upon Tyne, United Kingdom; 18 NIHR Exeter Clinical Research Facility, University of Exeter Medical School and Royal Devon and Exeter NHS Foundation Trust, Exeter, United Kingdom; 19 Department of Blood Sciences, Royal Devon and Exeter NHS Foundation Trust, Exeter, United Kingdom; 20 Department of Bio and Health Informatics, Technical University of Denmark, Kongens Lyngby, Denmark; 21 Novo Nordisk Foundation Center for Protein Research, Faculty of Health and Medical Sciences, University of Copenhagen, Copenhagen, Denmark; 22 Department of Genetic Medicine and Development, University of Geneva Medical School, Geneva, Switzerland; 23 Institute of Genetics and Genomics in Geneva (iGE3), University of Geneva, Geneva, Switzerland; 24 Swiss Institute of Bioinformatics, Geneva, Switzerland; 25 Affinity Proteomics, Science for Life Laboratory, School of Engineering Sciences in Chemistry, Biotechnology and Health, KTH—Royal Institute of Technology, Solna, Sweden; 26 Research Unit Molecular Endocrinology and Metabolism, Helmholtz Zentrum Muenchen, German Research Center for Environmental Health, Neuherberg, Germany; 27 Lehrstuhl für Experimentelle Genetik, Technische Universität München, Freising-Weihenstephan, Germany; 28 Department of Biochemistry, Yong Loo Lin School of Medicine, National University of Singapore, Singapore, Singapore; 29 HeLEX, Nuffield Department of Population Health, University of Oxford, Headington, Oxford, United Kingdom; 30 Division of Population Health & Genomics, School of Medicine, University of Dundee, Dundee, United Kingdom; 31 Department of Nutrition, Harvard School of Public Health, Boston, MA, United States of America; 32 Department of Biomedical Data Sciences, Molecular Epidemiology Section, Leiden University Medical Center, Leiden, The Netherlands; University of Pisa, ITALY

## Abstract

**Aim:**

Subclasses of different glycaemic disturbances could explain the variation in characteristics of individuals with type 2 diabetes (T2D). We aimed to examine the association between subgroups based on their glucose curves during a five-point mixed-meal tolerance test (MMT) and metabolic traits at baseline and glycaemic deterioration in individuals with T2D.

**Methods:**

The study included 787 individuals with newly diagnosed T2D from the Diabetes Research on Patient Stratification (IMI-DIRECT) Study. Latent class trajectory analysis (LCTA) was used to identify distinct glucose curve subgroups during a five-point MMT. Using general linear models, these subgroups were associated with metabolic traits at baseline and after 18 months of follow up, adjusted for potential confounders.

**Results:**

At baseline, we identified three glucose curve subgroups, labelled in order of increasing glucose peak levels as subgroup 1–3. Individuals in subgroup 2 and 3 were more likely to have higher levels of HbA1c, triglycerides and BMI at baseline, compared to those in subgroup 1. At 18 months (n = 651), the beta coefficients (95% CI) for change in HbA1c (mmol/mol) increased across subgroups with 0.37 (-0.18–1.92) for subgroup 2 and 1.88 (-0.08–3.85) for subgroup 3, relative to subgroup 1. The same trend was observed for change in levels of triglycerides and fasting glucose.

**Conclusions:**

Different glycaemic profiles with different metabolic traits and different degrees of subsequent glycaemic deterioration can be identified using data from a frequently sampled mixed-meal tolerance test in individuals with T2D. Subgroups with the highest peaks had greater metabolic risk.

## Introduction

Recent studies have shown significant heterogeneity in glucose response curves from oral glucose tolerance tests (OGTTs) in individuals with elevated risk of type 2 diabetes (T2D) [1–4]. Additionally, these glucose curves had distinct glycaemic risk profiles and were associated with future disease risk. Whether such distinct glycaemic profiles can also be identified in individuals with T2D has not been investigated to date. People with T2D differ in their rate of glycaemic deterioration and number and type of complications, indicating that T2D is a heterogeneous disease with different underlying pathophysiological mechanisms [5]. Therefore, studying the heterogeneity in glucose response curves and whether these glucose curves have distinct metabolic profiles may help improve our understanding of the different underlying pathophysiologies. We therefore aimed to examine the association between subgroups based on glucose curves during a five-point mixed-meal tolerance test (MMT) and metabolic traits at baseline and glycaemic deterioration in individuals with T2D.

## Materials and methods

### Study population

**Fig 1:**
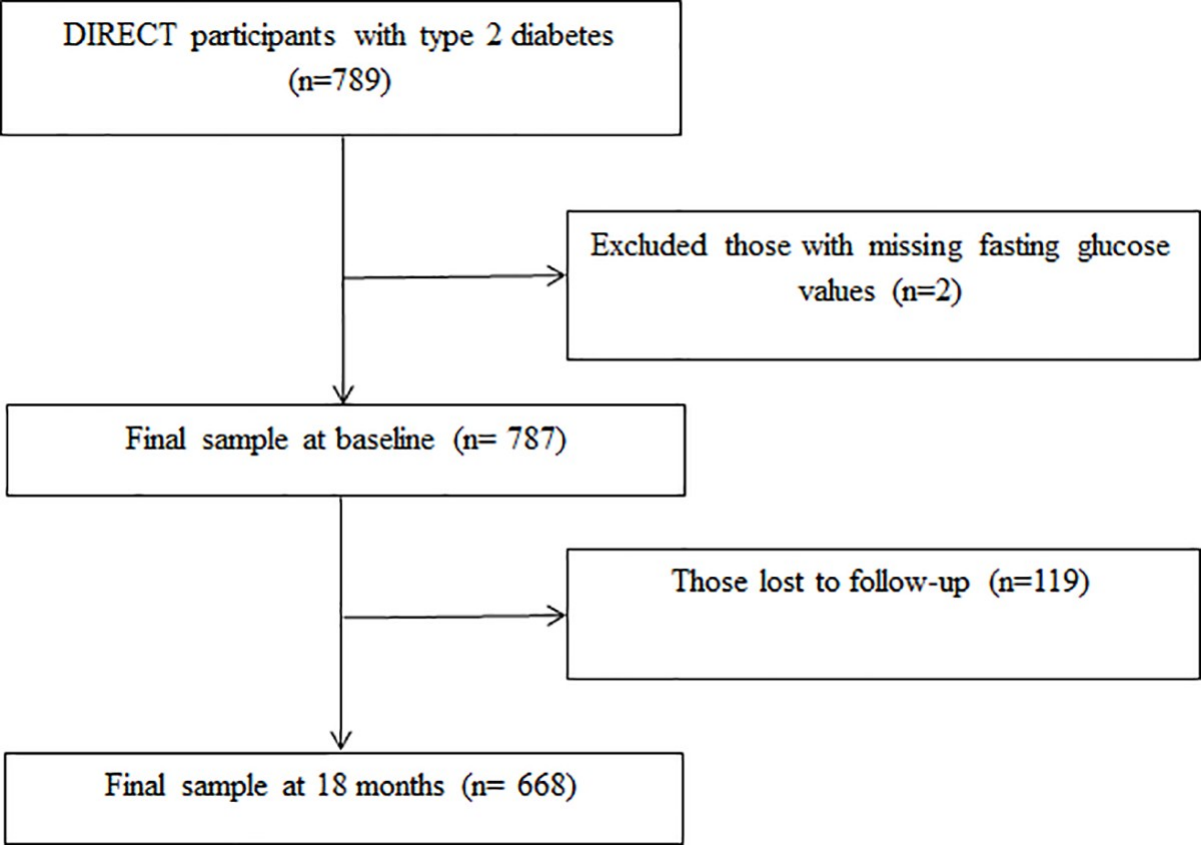
Flow chart of inclusion (n = 668) and exclusion (n = 221) of participants in the DIRECT study.

We used data from the Innovative Medicines Initiative-Diabetes Research on Patient Stratification (IMI-DIRECT) Study, which is described in detail elsewhere [6]. In brief, 789 individuals diagnosed with T2D within 6 to 24 months were recruited from six European clinical centers. Individuals were only recruited at baseline if they were using metformin therapy or on lifestyle management. If participants started using any diabetes medication or changed, this information was recorded at the next study visit. All participants signed informed consents and the IMI-DIRECT’s Data Access Committee—responsible for reviewing, approving and enabling access to data—approved this study. The study also conformed to the Declaration of Helsinki standards. We excluded those missing fasting glucose at baseline (n = 2) and those lost to follow-up (n = 119), thus 787 and 668 individuals with T2D were included in the analysis at baseline and follow-up respectively. At follow-up, MMT subgroups were identified in 651 individuals after excluding those missing fasting glucose (n = 17) (Fig 1). All individuals were white European adults, aged 35 to 75 years.

### MMT assessment

MMTs were performed at baseline and at 18 months after a 10-hour overnight fast. Blood was sampled at 0, 30, 60, 90 and 120 min. Participants were asked to stop the use of metformin, when used, 24 hours before the study visit. Participants consumed a 250ml liquid drink (Fortisip; 18.4 g carbohydrate per 100 ml) over a period of 2–5 min. Plasma glucose (mmol/l) was determined using the enzymatic glucose hexokinase method (Konelab 20 XT Clinical Chemistry analyzer, Thermo Fisher Scientific, Vantaa, Finland). C-peptide (nmol/l) and plasma insulin (pmol/l) were determined using chemiluminometric immunoassay (Liaison Insulin and Liaison C-peptid, DiaSorin S.p.A, Saluggia, Italy). HbA_1c_ (mmol/mol) was measured by ion-exchange high performance liquid chromatography on a TOSOH G8 analyser (Tosoh Bioscience, Inc, CA, USA). Triglycerides, total and high density lipoprotein (HDL) cholesterol were enzymatically assessed. More details on sample handling and biomarker determination are described elsewhere [6].

### Assessment of covariates

Questionnaires were used to collect data on age, sex, parental diabetes status, disease history, study center, smoking status, alcohol consumption and medication for diabetes and other conditions. Additionally, waist circumference (cm), BMI (kg/m^2^), blood pressure (mmHg) and physical activity (mgs) were determined at baseline and follow-up.

### Data analysis

Latent class trajectory analysis (LCTA) with cubic polynomials for the specification of time was used to identify glucose curve subgroups from MMT at baseline and at follow-up. The latent class mixed-effects models (lcmm) package in R (version 3.2.1) was used to conduct the LCTA. A random slope and random intercept were specified in the lcmm procedure. A random intercept was included to account for the correlation of measurements from the same individual. Optimal number of groups at baseline and follow-up was delineated by adding one more latent group at a time. To account for the chance of convergence to local maxima, the LCTA procedure was embedded in a gridsearch function. The best-fitting classification model was determined based on the Bayesian Information Criterion (BIC) and the Akaike Information Criterion (AIC). The lowest BIC and AIC suggesting the best fit and a difference of at least 10 points was regarded as sufficient improvement. The individuals were then assigned to a particular group based on their highest membership probability. Additionally, all mean membership probabilities for each class should be > 0.8 and this was also used to select the final number of groups. Furthermore, we produced plots of serum insulin levels corresponding to each subgroup’s glucose response curve at baseline.

We used general linear models to calculate coefficients (β) and 95% confidence intervals (CI) to estimate the association of identified subgroups with baseline metabolic traits and change in these metabolic traits at 18 months. We adjusted the p-values for multiple testing using the Bonferroni correction method.

Two multivariable models were formulated for the prospective analyses. Model 1 was adjusted for age, sex, follow-up, study center and respective baseline metabolic traits. Model 2 was additionally adjusted for smoking status, physical activity, family history of diabetes, diabetes duration and diabetes medication. Missing values were below 5% for all covariates with the exception of physical activity (8.4%). Therefore, we did a single imputation for physical activity values using the predictive mean matching method in the MICE (Multivariate Imputation by Chained Equations) package in R [7].

Furthermore, we compared baseline metabolic traits across individuals moving from subgroups at baseline to similar, lower or higher peak subgroups at follow-up.

For sensitivity analyses, we identified the baseline glucose curve subgroups stratified by study center and sex.

## Results

The analysis included 787 individuals at baseline; 58% men, age 62.1 ±8.1, BMI 30.5 ±5.0 kg/m^2^ and HbA1c 46.5 ±5.8 mmol/mol (6.2 ±0.5%) (Table 1).

**Table 1:** Characteristics of 787 individuals with type 2 diabetes stratified by glucose curve groups.

Characteristic	Glucose curve groups^1^		
	Subgroup 1	Subgroup 2	Subgroup 3
Number of participants	139	466	182
Age (years)	61.3 (7.5)	62.2 (8.0)	61.1 (8.6)
Sex, men [n]	87 (63%)	272 (58%)	97 (53%)
BMI (kg/m^2^)	30.0 (5.0)	30.9 (5.0)	30.2 (4.8)
Waist circumference (cm)	101.4 (13.5)	104.3 (13.3)	102.2 (12.8)
Smoking Status *Current* [n]	21 (15%)	65 (14%)	20 (11%)
Alcohol Status *Never* [n]	26 (19%)	71 (15%)	35 (19%)
Physical activity (mgs)	37.6 (11.2)	33.8 (9.3)	33.8 (9.8)
Systolic blood pressure (mmHg)	127.5 (15.4)	131.1 (15.0)	133.5 (17.0)
Diastolic blood pressure (mmHg)	74.6 (9.3)	75.2 (9.7)	76.5 (9.0)
HbA1c (mmol/mol)	43 (5)	46 (5)	50 (6)
HbA1c (%)	6.1 (0.4)	6.4 (0.5)	6.7 (0.6)
C-peptide (pmol/l)	908.6 (303.7)	1117.9 (416.9)	1122.2 (375.9)
Total cholesterol (mmol/l)	4.2 (1.4)	4.2 (1.1)	4.4 (1.1)
LDL (mmol/l)	2.4 (1.1)	2.3 (0.9)	2.4 (1.0)
HDL (mmol/l)	1.2 (0.4)	1.2 (0.4)	1.2 (0.4)
Triglycerides (mmol/l)	1.1 [0.9,1.5]	1.4 [1.0,1.9]	1.5 [1.1,2.0]
Fasting insulin (pmol/l)	83.8 (54.9)	110.5 (74.3)	113.8 (69.8)
Glucose peak values (mmol/l)^2^	8.4	10.0	13.1
2h postprandial insulin (pmol/l)	150.7 [99.8,258.5]	405.8 [253.4,615.0]	463.4 [321.7,624.1]
Fasting plasma glucose (mmol/l)	6.4 (1.3)	7.1 (1.2)	7.9 (1.7)
1h postprandial glucose(mmol/l)	8.0 (2.3)	10.0 (2.1)	12.3 (2.5)
2h postprandial glucose(mmol/l)	5.0 (1.3)	8.3 (1.6)	12.3 (1.9)
Diabetes duration	1.2 (0.9)	1.7 (6.6)	1.8 (5.4)
Diabetes meds at baseline (metformin) *Yes* [n]	32 (23%)	158 (35%)	82 (45%)
Changed diabetes meds during follow-up *Yes* [n]	12 (8%)	81 (17%)	61 (35%)
Center [n (row %)]			
*Copenhagen*	10 (19)	25 (48)	17 (33)
*Lund*	25 (26)	58 (60)	13 (14)
*Newcastle*	12 (9)	84 (60)	45 (32)
*Exeter*	32 (23)	103 (62)	31 (15)
*Dundee*	35 (19)	100 (62)	31 (19)
*Hoorn*	25 (15)	96 (57)	45 (27)
Family history, parents *Yes* [n(%)]	55 (40)	168 (36)	67 (37)

^1^ Mean ± SD for continuous data and all such values unless stated otherwise.

^2^ We assessed the highest glucose value in each subgroup as the peak.

Abbreviations: BMI: body mass index; 2h:2 hour; LDL: low density lipoproteins; HDL: high density lipoproteins.

**Fig 2:**
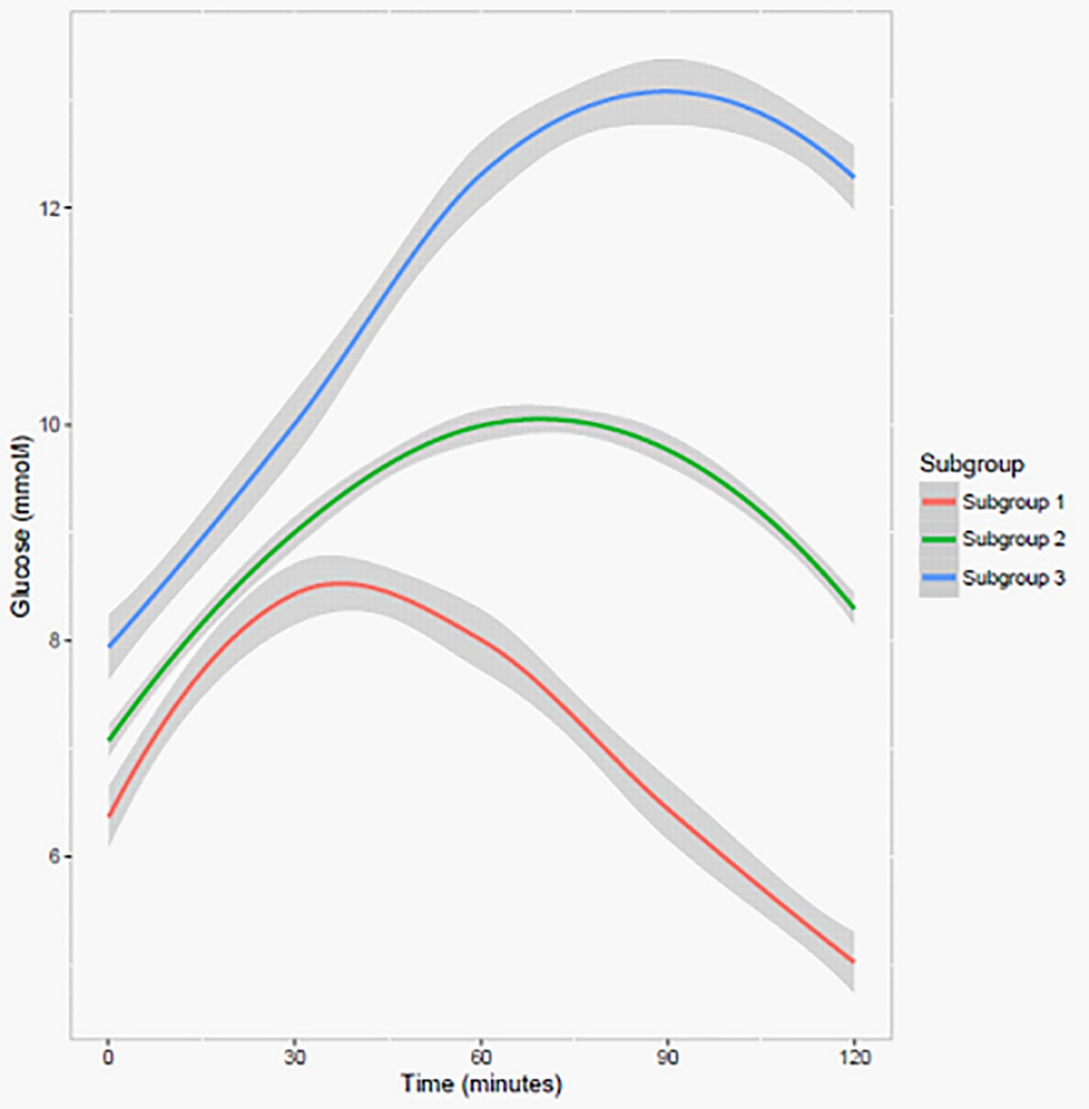
Glucose curve subgroups following a MMT depicting estimated mean trajectories with 95% confidence intervals identified by the latent class trajectory analysis in individuals with type 2 diabetes (n = 787).

We identified three subgroups with different glucose patterns following an MMT, labelled in order of increasing glucose peak levels as subgroup 1–3 (Fig 2), consisting of 139 (18%), 466 (59%) and 182 (23%) individuals, respectively (Table 1). Subgroup 1 had the earliest and lowest glucose peak (8.4 mmol/l), while subgroup 3 took the longest to reach peak and had the highest glucose peak (13.1 mmol/l), and subgroup 2 had a glucose peak of 10.0 mmol/l. Membership probabilities were all above 0.80, ranging from 0.86 to 0.90. The four subgroup solution met all but one of the selection criteria, one of the membership probabilities was below 0.8 (S1 Fig and Table 2). Stratified by sex and center, three similar subgroups were identified. Heterogeneity was also evident in the insulin response curve subgroups. Subgroup 1 had the earliest and lowest insulin peak with the lowest 2-hour insulin value, while subgroup 3 had the latest insulin peak and highest 2-hour value (S2 Fig). Individuals in higher glucose peak subgroups i.e. subgroup 2 and 3, were more likely to have higher metabolic trait values such as higher BMI, insulin and glucose values compared to individuals in subgroup 1 (Table 1) and hence a less favourable metabolic risk profile compared to individuals in subgroup 1.

**Table 2:** Bayesian and Akaike Information Criterion and mean posterior probability values.

	Highest mean posterior probabilities in each class >80%					
Number of classes	BIC	AIC	Class 1	Class 2	Class 3	Class 4
1-class	14952.48	14910.47				
2-class	14633.14	14567.79	0.90	0.90		
3-class	14535.27	14446.57	0.86	0.89	0.88	
4-class	14393.16	14505.20	0.85	0.82	0.87	0.75

Abbreviations: BIC: Bayesian Information Criterion; AIC: Akaike Information Criterion.

At 18 month follow-up, the beta-coefficients (95% CI) for change in HbA1c (mmol/mol) significantly increased across subgroups with 0.37 (-0.18–1.92) for subgroup 2 and 1.88 (-0.08–3.85) for subgroup 3, relative to subgroup 1 (Table 3). The same trend was observed for change in triglycerides and fasting glucose with the exception of HDL. Generally, individuals in subgroup 2 and 3 progressed faster in metabolic parameters than individuals in subgroup 1.

**Table 3:** Association between glucose curve groups and metabolic traits measured at 18 months in 668 individuals with type 2 diabetes.

Characteristics	Glucose curve groups^1^			
	Subgroup 1	Subgroup 2	Subgroup 3	P-Value
Number of participants	125	389	154	
Follow up (months)	18.2 (0.5)	18.2 (0.6)	18.3 (0.8)	
HBA1c (mmol/mol)	-			
Model 1	-	0.48 (-1.01,1.99)	2.66 (0.76,4.57)	**<0.001**
Model 2	-	0.37 (-0.18,1.92)	1.88 (-0.08,3.85)	**<0.001**
Triglycerides (mmol/l)^2^				
Model 1	-	0.09 (-0.06,0.24)	0.09 (-0.10,0.28)	**<0.001**
Model 2	-	0.10 (-0.06,0.25)	0.13 (-0.05,0.31)	**<0.001**
Total cholesterol (mmol/l)				
Model 1	-	-0.11 (-0.28,0.05)	-0.05 (-0.24,0.15)	**0.188**
Model 2	-	-0.10 (-0.27,0.19)	-0.02 (-0.23,0.44)	**0.227**
LDL (mmol/l)				
Model 1	-	-0.07 (-0.22,0.07)	-0.01 (-0.19,0.35)	**0.323**
Model 2	-	-0.07 (-0.22,0.08)	0.01 (-0.17,0.19)	**0.150**
HDL (mmol/l)				
Model 1	-	-0.09 (-0.14,-0.04)	-0.11 (-0.17,-0.05)	**<0.001**
Model 2	-	-0.08 (-0.13,-0.03)	-0.09 (-0.15, 0.03)	**0.002**
Plasma insulin (pmol/l)^2^				
Model 1	-	-3.24 (-15.17,8.68)	-11.50 (-25.62,2.62)	**0.012**
Model 2	-	-5.06 (-17.85,7.73)	-15.88 (-31.08,0.68)	**0.250**
Fasting glucose (mmol/l)				
Model 1	-	0.12 (-0.19,0.43)	0.79 (0.39,1.18)	**<0.001**
Model 2	-	0.06 (-0.26,0.38)	0.59 (0.19,0.99)	**<0.001**

^1^ Values are Beta coefficients (95% confidence intervals).

^2^ Variables were log transformed before analysis.

Abbreviations: HbA1c: glycated haemoglobin; T2D: type 2 diabetes. Model 1 is adjusted for age, sex, study center, baseline values and follow up. Model 2 is adjusted for model 1 plus DM medication, family history of parents DM status, DM duration, physical activity and smoking status. P-values (significance level <0.05) remained robust after Bonferroni correction for multiple testing.

**Fig 3:**
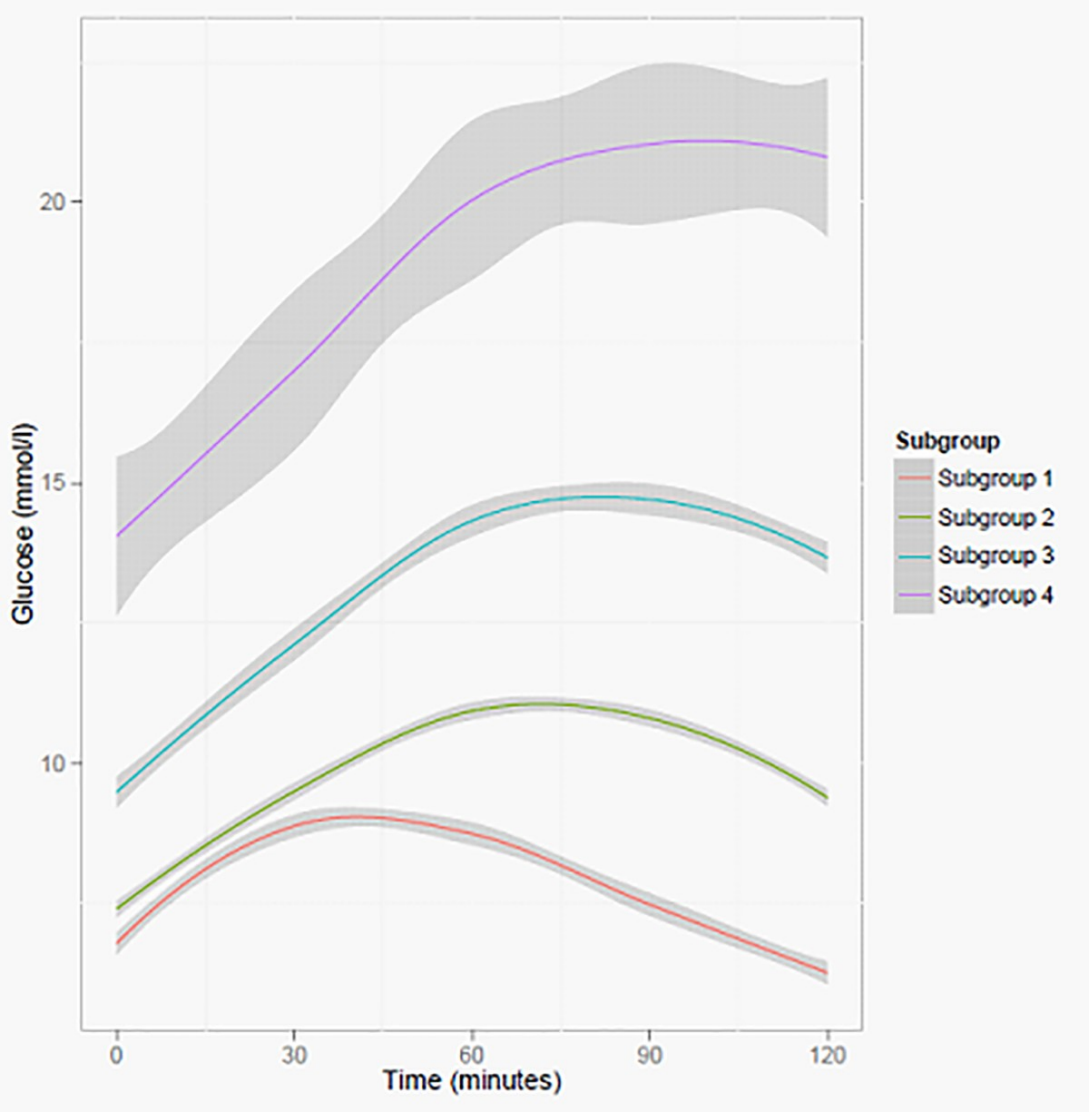
Glucose curve subgroups following a MMT depicting estimated mean trajectories of the 4 group solution identified by the latent class trajectory analysis in 651 individuals with type 2 diabetes at 18 months of follow-up.

The optimum number of glucose curve subgroups identified at follow-up was four with the fourth subgroup having the highest peak, fasting and 2-hour glucose values (Fig 3). The percentage of individuals moving from subgroups at baseline to higher peak subgroups at follow-up was 38%, 15% and 7% for subgroup 1–3, respectively. Individuals from baseline subgroups moving to lower peak subgroups at follow-up were 27% and 46% for subgroup 2 and 3, respectively. In short, majority of participants in subgroups at baseline remained in the same subgroups at follow-up (Table 4). Individuals moving from baseline subgroups to higher peak subgroups at follow-up, were more likely to have higher BMI, waist circumference, diastolic pressure, hbA1c, fasting glucose and fasting insulin. They were also more likely to be males, use metformin and had the highest family history of T2D. Most of these characteristics including change in T2D medication were lowest in those moving from higher peak baseline subgroups to lower peak subgroups at follow-up (S1 Table).

**Table 4:** Comparisons of subgroups identified at baseline and at follow-up.

Baseline groups [n(row%)]	Follow-up groups			
	Subgroup 1	Subgroup 2	Subgroup 3	Subgroup 4
Subgroup 1	78 (62)	42 (34)	5 (4)	0 (0)
Subgroup 2	104 (27)	220 (57)	61 (16)	1 (0)
Subgroup 3	8 (6)	55 (40)	67 (48)	10 (7)

## Discussion

Using data from 787 individuals with T2D, who had undergone a frequently sampled MMT, combined with the LCTA approach, three glucose curve subgroups were identified. The individuals within these subgroups differed in their metabolic traits at baseline and their rates of subsequent glycaemic deterioration over 18 months. Individuals with T2D in the subgroups with the highest plasma glucose peaks and the highest 1-hour and 2-hour glucose levels (subgroups 2 and 3) had the worst metabolic traits profile, compared to individuals in the subgroup with the lowest and earliest glucose peak. Moreover, individuals moving from baseline subgroups to higher peak subgroups at follow-up had the worst metabolic traits profile compared to those moving from higher peak baseline subgroups to lower peak subgroups at follow-up.

To the best of our knowledge, there is no study that has exhaustively studied the heterogeneity in glucose curves following an MMT in individuals with T2D. However, several studies identified glucose response curves following an OGTT in those at elevated diabetes risk [1, 4, 8], and most of them identified four glucose response subgroups. The disparity in the number of subgroups between these studies and ours could be because we had a relatively more homogenous group of individuals with T2D (without insulin therapy) and hence less heterogeneity. Nevertheless, the subgroups with the highest peaks and 1-hour value in these studies had the worst metabolic risk profile which is in line with our findings.

The various plausible mechanisms that could explain the differences in the identified subgroups in our study include differences in insulin resistance and secretion. Subgroup 2 and 3 had higher BMI, insulin and glucose values, thereby indicating that they were more insulin resistant than those in subgroup 1. Subgroup 2 and 3 also had higher 1-hour glucose values compared to subgroup 1 and studies showed that higher 1-hour plasma glucose is associated with an increased risk of T2D complications [9–14]. High 1-hour glucose value could be a result of impaired early phase insulin secretion and reduced insulin sensitivity which could explain the heterogeneity we see in the subgroups.

Some limitations of our study include the limited generalizability of our results to other ethnicities due to the inclusion of only white European adults. Second, a short follow-up time of 18 months does not allow us to investigate associations with hard cardiovascular outcomes. Lastly, we did not have information on adherence to diabetes drugs which might influence progression within the 18 months period, however, we adjusted for the use of diabetes medication to try and mitigate this. Our study was strengthened by the use of a large sample with elaborate metabolic parameters at baseline and follow up.

In conclusion, different glycaemic profiles with different metabolic traits and different degrees of subsequent glycaemic deterioration can be identified using data from a frequently sampled MMT in individuals with T2D. This heterogeneity in glucose curves suggests different underlying pathophysiologies. However, more similar studies should be done to confirm the robustness of these results.

## Supporting information

S1 Fig Glucose curve subgroups following a MMT depicting estimated mean trajectories of the 4 group solution identified by the latent class trajectory analysis in 787 individuals with type 2 diabetes at baseline. (DOCX)

S2 Fig Insulin curve subgroups corresponding to the 3 glucose curve groups following a MMT depicting estimated mean trajectories identified by the latent class trajectory analysis in 787 individuals with type 2 diabetes at baseline. (DOCX)

S1 Table Characteristics of 651 individuals stratified by movement from baseline subgroups to similar, lower or higher peak subgroups at follow-up. (DOCX)

## Abbreviations

BIC: Bayesian Information Criterion
AIC: Akaike Information Criterion
IMI: Innovative Medicines Initiative
DIRECT: Diabetes Research on Patient Stratification
LCMM: Latent class mixed models
LCTA: Latent class trajectory analysis
MICE: Multivariate Imputation by Chained Equations
MMT: Mixed-meal tolerance
