# Supplementary material for: Post-load glucose subgroups and associated metabolic traits in individuals with type 2 diabetes: An IMI-DIRECT study
Source: PLoS One. 2020 Nov 30;15(11):e0242360. doi: 10.1371/journal.pone.0242360 (PMC7703960; doi:10.1371/journal.pone.0242360)
Supplement: S1 Fig — (DOCX) [file pone.0242360.s001.docx]

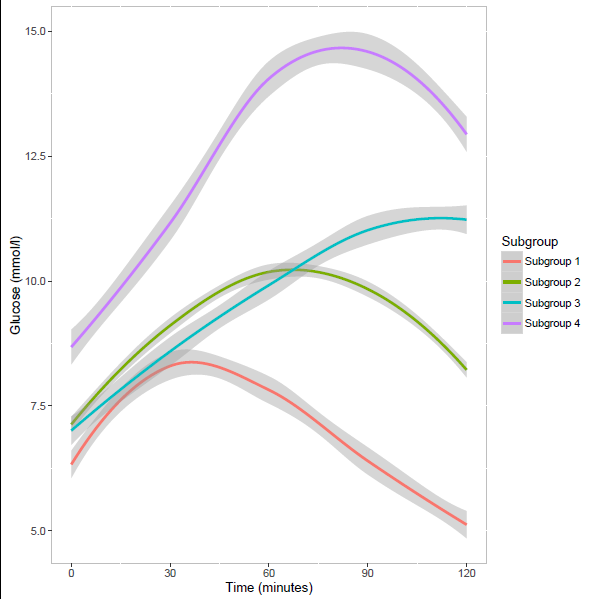


S1 Fig. Glucose curve subgroups following a MMT depicting estimated mean trajectories of the 4 group solution identified by the latent class trajectory analysis in 787 individuals with type 2 diabetes at baseline.
