# Supplementary material for: Post-load glucose subgroups and associated metabolic traits in individuals with type 2 diabetes: An IMI-DIRECT study
Source: PLoS One. 2020 Nov 30;15(11):e0242360. doi: 10.1371/journal.pone.0242360 (PMC7703960; doi:10.1371/journal.pone.0242360)
Supplement: S1 Table — (DOCX) [file pone.0242360.s003.docx]

**S1 TABLE Characteristics of 651 individuals stratified by movement from baseline subgroups to similar, lower or higher peak subgroups at follow-up.**

| Characteristic | Movement of glucose curve groups^1^ | | |
| --- | --- | --- | --- |
|  | Similar | High to low | Low to high |
| Number of participants  Age (years)  Sex, men [n]  BMI (kg/m^2^)  Waist circumference (cm)  Smoking Status *Current* [n]  Alcohol Status *Never* [n]  Physical activity (mgs)  Systolic blood pressure (mmHg)  Diastolic blood pressure (mmHg)  HbA1c (mmol/mol)  HbA1c (%)  Fasting insulin (pmol/l)  Fasting plasma glucose (mmol/l)  Diabetes meds at baseline(metformin) *Yes* [n]  Changed diabetes meds at follow-up *Yes* [n]  Family history, parents *Yes* [n] | 365  62.6 (7.5)  219 (60%)  30.4 (4.8)  102.7 (12.9)  40 (11%)  60 (16%)  34.2 (9.6)  130.9 (16.5)  74.8 (10.0)  47 (6)  6.4 (0.5)  103.4 (68.8)  7.1 (1.4)  131 (36%)  92 (25%)  139 (38%) | 167  62.3 (8.0)  98 (59%)  29.7 (4.9)  100.9 (13.4)  21 (13%)  22 (13%)  34.9 (9.2)  131.6 (15.4)  74.1 (9.6)  46 (6)  6.3 (0.5)  98.0 (60.6)  7.0 (1.2)  50 (30%)  33 (20%)  57 (34%) | 119  61.4 (7.9)  75 (63%)  31.4 (5.4)  105.5 (14.4)  15 (13%)  16 (13%)  36.3 (12.0)  130.0 (13.9)  77.2 (8.7)  48 (6)  6.5 (0.6)  113.7 (77.6)  7.2 (2.0)  45 (38%)  28 (24%)  48 (40%) |

^1^ Mean ± SD for continuous data and all such values unless stated otherwise. Abbreviations: BMI: body mass index.
